# Fluctuation of physical function during chimeric antigen receptor T‐cell therapy during rehabilitation intervention: Real‐world data and risk factor analyses

**DOI:** 10.1002/jha2.1043

**Published:** 2024-11-04

**Authors:** Ryota Hamada, Yasuyuki Arai, Toshio Kitawaki, Naokazu Nakamura, Masanobu Murao, Michiko Matsushita, Junsuke Miyasaka, Tsugumi Asano, Tomoyasu Jo, Momoko Nishikori, Junya Kanda, Chisaki Mizumoto, Kouhei Yamashita, Ryosuke Ikeguchi, Akifumi Takaori‐Kondo

**Affiliations:** ^1^ Department of Rehabilitation Kyoto University Hospital Kyoto Japan; ^2^ Department of Hematology Kyoto University Hospital Kyoto Japan; ^3^ Center for Research and Application of Cellular Therapy Kyoto University Hospital Kyoto Japan

**Keywords:** CAR‐T, physical function, rehabilitation

## Abstract

**Introduction:**

Patients undergoing chimeric antigen receptor (CAR) T‐cell therapy face prolonged treatment timelines and are prone to cytokine release syndrome (CRS) and immune effector cell‐associated neurotoxicity syndrome (ICANS) after infusion. Disabilities in physical function and the importance of rehabilitation during CAR‐T‐cell therapy to maintain physical function have been poorly documented.

**Method:**

We performed a retrospective cohort study to assess changes in exercise tolerance via differences in a 6‐min‐walking distance (Δ6MWD) and factors influencing it.

**Results:**

A total of 77 patients who underwent rehabilitation during CAR‐T‐cell therapy were enrolled, and their 6MWD was 450 m (median, range 180–705 m) before and 450.5 m (107.0–735.0 m) 30 days after CAR‐T treatment. No significant alteration in Δ6MWD was observed overall (11.0 m, 95% confidence interval, −56.1 to 88.2 m). Multiple regression analyses indicated that age (over vs. under 65 years) revealed no notable differences in Δ6MWD (20 vs. 10 m), while ΔHb (*β* = 0.24, *p* = 0.03), moderate/severe CRS (grade 1 with continuous fever or grade ≥2; *β* = −0.25, *p* = 0.03), and ICANS (any grade; *β* = −0.22, *p* = 0.04) were significantly associated with lower Δ6MWD.

**Conclusion:**

This real‐world study indicated that CAR‐T‐cell therapy is less likely to reduce physical function even in older patients if rehabilitation is properly performed, whereas CRS and ICANS can be risk factors to deprive exercise tolerance.

## 1 INTRODUCTION

Chimeric antigen receptor (CAR) T‐cell therapy is one of the most efficacious treatment strategies for some relapsed and refractory hematopoietic malignancies. Among various complications related to this treatment, cytokine release syndrome (CRS) and immune effector cell‐associated neurotoxicity syndrome (ICANS) are major adverse events that can impact the early post‐treatment phase. In most cases, CRS and ICANS can be controlled safely with tocilizumab and steroids [1, 2] and are rarely life‐threatening, but their impacts on later physical functions are still unknown.

Some multicenter studies have reported the prevalence of moderate‐to‐severe fatigue after CAR‐T treatment, prompting discussions on the necessity of rehabilitation [3, 4], but quantitative assessments of physical functions of these patients before and after CAR‐T treatment remain unreported [5]. Therefore, it is difficult for physicians to judge the utility of this treatment, especially for frail and/or elderly patients, although other patients can generally tolerate CAR‐T more easily than autologous or allogeneic stem cell transplantation, because conditioning intensity (lymphocyte depletion chemotherapy vs. high‐dose chemotherapy and/or total body irradiation) and bone marrow suppression after treatments are more modest [6].

In this study, we performed retrospective real‐world data analyses to (1) investigate changes in exercise tolerance before and after treatment among CAR‐T‐cell therapy patients undergoing rehabilitation intervention, and to (2) identify risk factors for deterioration of physical function, focusing mainly on age and CAR‐T‐specific complications. These results may be important to determine eligibility for CAR‐T treatment and to investigate appropriate rehabilitation interventions during CAR‐T‐cell therapy.

## 2 MATERIALS AND METHODS

### 2.1 Patients and data collection

This retrospective observational study focused on adult patients who underwent CAR‐T‐cell therapy with rehabilitation interventions at Kyoto University Hospital between 2020 January and 2023 December. All patients were treated at admission from the beginning of lymphocyte depletion chemotherapy to around 3 weeks after CAR‐T infusion. The study adhered to principles outlined in the Declaration of Helsinki and was approved by the pertinent ethics committee in Kyoto University (approval number: R‐0715).

Clinical parameters used in this study included Eastern Cooperative Oncology Group performance status (ECOG‐PS), hematopoietic cell transplantation comorbidity index (HCT‐CI), hemoglobin (Hb) levels, lactate dehydrogenase (LDH) levels, and disease status at infusion, classified as complete response (CR), partial response (PR), stable disease (SD), or progressive disease (PD). Additionally, we included details regarding the CAR‐T‐cell therapy product type (tisa‐cel, liso‐cel, axi‐cel, and ide‐cel) and participant treatment histories previous to CAR‐T‐cell therapy, such as the number of prior lines of therapy, prior treatment courses, and history of allogeneic or autologous stem cell transplantation. In myeloma cases, a very good partial response (VGPR) was considered within the PR category for analysis, in alignment with lymphoma cases. Evaluation of the post‐CAR‐T treatment course included an assessment of complications, including CRS and ICANS, with severities, durations, and the types of treatment.

### 2.2 Management of complications after CAR‐T‐cell therapy

Complications following CAR‐T‐cell therapy, including diagnosis and severity of CRS and ICANS, were assessed in accordance with guidelines from the American Society for Transplantation and Cell Therapy [7]. Furthermore, these assessments aligned with appropriate guidelines for specific CAR‐T‐cell therapy products (tisa‐cel [Kymriah; Novartis], liso‐cel [Breyanzi; Bristol Myers Squibb], axi‐cel [Yescarta; Kite Pharma], and ide‐cel [Abecma; Bristol Myers Squibb]).

Severity of CRS was categorized using the ASTCT CRS grade [7] and the modified CRS (m‐CRS) grade [8]. This modified grading system further stratifies grade 1 CRS into grades 1a and 1b, based on the duration of CRS‐related fever, aiming for a more nuanced assessment of CRS severity over time. Grade 1a was defined as CRS grade 1 for <5 days, whereas grade 1b ≥5 days.

### 2.3 Rehabilitation and exercise tolerance assessment

Participants in this study began rehabilitation interventions on the day they were admitted for CAR‐T‐cell therapy. Patients received exercise therapy five times per week (20–40 min per session) throughout their hospitalization. Exercise therapy, which consisted of stretching, strength training, and aerobic training, and exercise intensity, was designed to achieve a subjective exercise intensity of 4 (somewhat strenuous) on the modified Borg scale. Even when severe complications necessitated intensive care, rehabilitation was continued as much as possible through close coordination with doctors and nurses.

The 6‐min walk test was used to assess exercise tolerance [9, 10], according to the protocol recommended by the American Thoracic Society [11]. The 6‐min walking distance (6MWD) under maximum effort was measured and evaluated as the index for exercise tolerance. This assessment was performed before treatment (before lymphocyte depletion chemotherapy) and after treatment (1 month after infusion), and the change in 6MWD before and after treatment (Δ6MWD) was calculated.

### 2.4 Statistical analysis

Statistical analyses were performed using SPSS version 29.0 (IBM Corp.). The Shapiro–Wilk test was used to assess data distribution. Comparisons of 6MWD values before and after treatment across the entire subject population were analyzed using the corresponding *t*‐test. Subsequently, comparisons of the pre‐ and post‐treatment 6MWD within each subgroup were made based on sex (male vs. female), age (<65 vs. ≥65 years), diagnosis (lymphoma vs. multiple myeloma), disease status (CR/PR vs. SD/PD), CRS severity (grade ≤1a vs. grade ≥1b), and ICANS occurrence. The Mann–Whitney *U*‐test was used to compare the Δ6MWD between groups.

Moreover, Spearman's correlation coefficient was calculated to examine the association between pre‐CAR‐T 6MWD and clinical status pre‐CAR‐T (including prior chemotherapy, HCT‐CI, LDH levels, and disease status), as well as the association of Δ6MWD with CRS‐related fever duration and changes in Hb before and after treatment (ΔHb).

Furthermore, multiple regression analysis was conducted to identify factors influencing Δ6MWD before and after treatment among CAR‐T‐cell therapy patients. Statistical significance was defined as *p* < 0.05.

## 3 RESULTS

### 3.1 Patient characteristics

Eighty patients received CAR‐T‐cell therapy, but three patients were not enrolled in this study because of missing data on 6MWD (due to bone fragility, impaired consciousness, or early death after CAR‐T‐cell therapy). Therefore, 77 patients (39 male and 38 female) with a median age of 62 years and a median body mass index (BMI) of 21.2 kg/m^2^ were included. CAR‐T products administered included tisa‐cel in 48 patients, liso‐cel in 18 patients, axi‐cel in four patients, and ide‐cel in seven patients.

Before treatment, 26 patients (34%) exhibited an ECOG‐PS score of 1 or greater, whereas 19 patients (25%) had comorbidities with a score greater than 1 on HCT‐CI. The predominant disease type was malignant lymphoma, and seven patients were treated with CAR‐T for multiple myeloma. Median numbers of treatment lines and courses prior to CAR‐T‐cell therapy were 4 and 12, respectively. Allogeneic or autologous transplantation before CAR‐T was performed in 26 patients. Regarding disease status at the time of CAR‐T‐cell infusion, 24 patients (31%) were in CR, 29 patients (38%) were in PR, 13 patients (17%) had SD, and 11 patients (14%) showed PD. Other patient characteristics are shown in Table 1.

**TABLE 1:** Patient characteristics.

	Total (*N *= 77)
Age, year	62 (20–75)
Sex, male/female	39/38
BMI, kg/m^2^	21.2 (13.8–31.5)
ECOG‐PS, 0/1/2/3	51/21/4./1
HCT‐CI, 0–1/2–	58/19
Diagnosis, DLBCL/FL/MM	63/3/7
Hb, g/dL	8.9 (6.2–14)
LDH, U/L	245 (133–652)
Disease status at infusion, CR/PR/SD/PD	24/29/13/11
Number of lines, *n*	4 (2–11)
Number of courses, *n*	12 (5–52)
History of AlloSCT, *n*	4
History of ASCT, *n*	22
CAR‐T product, tisa‐cel/liso‐cel/axi‐cel/ide‐cel, *n*	48/18/4/7

Data are presented as median (range) unless otherwise indicated.

Abbreviations: ASCT, autologous stem cell transplantation; BMI, body mass index; CAR, chimeric antigen receptor; CR, complete response; DLBCL, diffuse large B‐cell lymphoma; ECOG PS, Eastern Cooperative Oncology Group performance status; FL, follicular lymphoma; Hb, hemoglobin; HCT‐CI, hematopoietic cell transplantation comorbidity index; LDH, lactate dehydrogenase; MM, multiple myeloma; PD, progressive disease; PR, partial response; SD, stable disease.

### 3.2 Clinical courses after CAR‐T‐cell therapy

Among study participants, 71 (92%) experienced CRS with 62 patients (87%) in grade 1, eight patients (11%) in grade 2, and one patient (1%) in grade 3 according to ASTCT grade. Median duration of CRS‐related fever was 5 days (range, 1–18), and grade 1 patients were regraded as grade 1a (31 patients) or grade 1b (31 patients) according to the m‐CRS grading system. Tocilizumab was administered to 58 patients (75%) and steroids were used with 22 patients (29%) as part of the CRS treatment. ICANS was documented in seven patients (9%). Intensive care management was necessary in 16 patients with these complications (Table 2).

**TABLE 2:** Course of treatment.

	Total (*N *= 77)
CRS, +/−, *n*	71/6
CRS grade, 1/2/3, *n*	62/8/1
m‐CRS grade, 1a/1b/2/3, *n*	31/31/8/1
Fever duration associated with CRS, day	5 (1–18)
Tocilizumab, dose/nondose	58/19
ICANS, +/−, *n*	7/70
Steroid, dose/nondose	22/55

Abbreviations: CRS, cytokine release syndrome; ICANS, immune effector cell‐associated neurotoxicity syndrome.

### 3.3 Change in exercise tolerance pre‐ and post‐CAR‐T‐cell therapy

**FIGURE 1:**
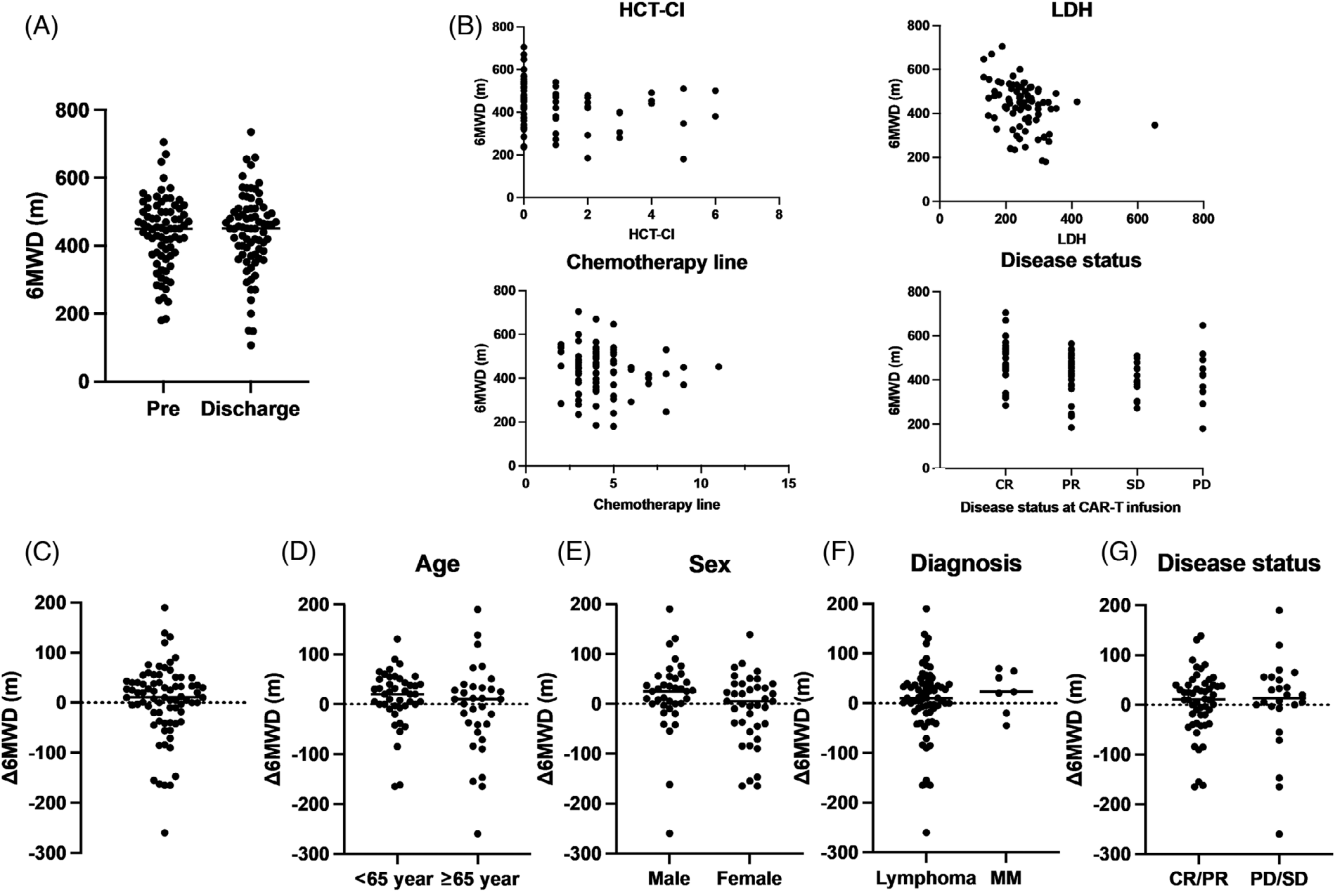
Six‐min‐walking distance (6MWD) before and after chimeric antigen receptor (CAR) T‐cell therapy. (A) 6MWD before and after treatment for all CAR‐T‐cell therapy patients. (B) Association of 6MWD with background prior to CAR‐T‐cell therapy. (C) Distribution of Δ6MWD among CAR‐T‐cell therapy patients. Distribution of Δ6MWD among CAR‐T‐cell therapy patients classified by (D) age 65, (E) sex, (F) diagnosis, and (G) disease status. CR, complete response; HCT‐CI, hematopoietic cell transplantation comorbidity index; LDH, lactate dehydrogenase; MM, multiple myeloma; PD, progressive disease; PR, partial response; SD, stable disease

First, we checked 6MWD prior to CAR‐T and its association with various clinical factors. Pre‐CAR‐T 6MWD ranged from 180.0 to 705.0 m (450.0 m in median; Figure 1A). Significant associations were observed with HCT‐CI (*r* = ‐0.23, *p* = 0.03), LDH levels (*r* = ‐0.34, *p* < 0.01), and disease status at CAR‐T infusion (*r* = ‐0.24, *p* = 0.03), while prior chemotherapy (lines: *r* = ‐0.14, *p* = 0.22; courses: *r* = ‐0.11, *p* = 0.32) was not associated (Figure 1B).

Next, we focused on changes in 6MWD. 6MWD in the whole cohort demonstrated no significant change before and after CAR‐T‐cell therapy (median 450.0 m before treatment, 450.5 m after treatment, *p* = 0.68). The difference of 6MWD in each patient ranged from −260.0 to +190.0 m (median, +11.0 m), and this, too, was statistically insignificant (Figure 1C).

Subsequently, we performed several subgroup analyses (for age, sex, diagnosis, and disease status) to identify variables closely related to Δ6MWD. There was no significant difference in Δ6MWD between sexes (+25 m in male vs. +5 m female group; *p* = 0.08; Figure 1D), in age at CAR‐T‐cell therapy (+20 m in <65 years group vs. +10 m ≥65 years group; *p* = 0.19; Figure 1E), between diagnosis (+10 m in lymphoma vs. +23 m  multiple myeloma group; *p* = 0.45; Figure 1F), or in disease status at CAR‐T‐cell therapy (+11 m in CR/PR group vs. 0 m PD/SD group; *p* = 0.77; Figure 1G).

### 3.4 Association with exercise tolerance and CAR‐T‐specific complications

**FIGURE 2:**
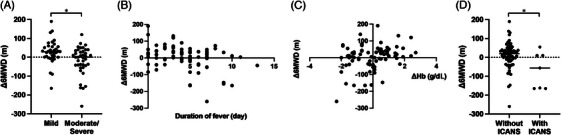
Complications and changes in 6‐min‐walking distance (6MWD) after chimeric antigen receptor (CAR) T‐cell therapy. (A) Distribution of Δ6MWD among CAR‐T‐cell therapy patients classified by m‐cytokine release syndrome (CRS) grade. (B) Relationship between Δ6MWD and CRS‐related fever duration. (C) Relationship between Δ6MWD and ΔHb, (D) Range of Δ6MWD among CAR‐T‐cell therapy patients classified by immune effector cell‐associated neurotoxicity syndrome (ICANS).

Regarding CRS, Δ6MWD showed a significant difference when analyzed using conventional CRS grading (+20.0 m in CRS grade 0–1 vs. ‐37.0 m in grade 2–, *p* = 0.02). However, this analysis is not statistically robust due to the skewed number of patients in each subcohort (68 patients in CRS grade 0–1 vs. nine patients in grade 2–). Therefore, we performed the same analysis using m‐CRS grade between mild CRS (patients with grade 1a or lower, *N* = 37) and moderate/severe CRS (patients with grade 1b or higher, *N* = 40). We found significant differences in Δ6MWD between these groups (+29.0 m in mild CRS vs. 0.0 m in moderate/severe CRS, *p* < 0.01; Figure 2A). There was a significant negative correlation between Δ6MWD and the duration of CRS‐related fever (*r* = ‐0.35, *p* < 0.01; Figure 2B), as well as a significant positive correlation between ΔHb and Δ6MWD (*r* = 0.28, *p* = 0.01; Figure 2C). Moreover, patients who developed ICANS showed significantly decreased Δ6MWD (+10.6 m in patients without ICANS vs. −67.5 m in patients with ICANS, *p* = 0.02; Figure 2D), although ICANS was observed in relatively few patients (*N* = 7, 9%).

### 3.5 Multivariate analyses for factors influencing exercise tolerance in CAR‐T‐cell therapy patients

According to results shown above (univariate analyses), we performed multivariate analyses, including pre‐CAR‐T patient characteristics (sex, age, and disease status). CAR‐T‐related variables (ΔHb, m‐CRS grade, and ICNAS) indicated that ΔHb (*β* = 0.24, *p* = 0.03), moderate/severe m‐CRS (*β* = ‐0.25, *p* = 0.03), and development of ICANS (*β* = ‐0.22, *p* = 0.04) showed significant factors negatively associated with Δ6MWD (Table 3). These results indicated that the patient cohort with moderate/severe CRS and associated cytopenia/or ICANS experienced a significant decrease or delayed recovery in 6MWD after CAR‐T treatment, suggesting weakened physical tolerance during CAR‐T treatment.

**TABLE 3:** Multiple regression analysis to identify factors influencing e6‐min‐walking distance (Δ6MWD).

Adjusted *R* ^2^	Variables	Partial regression coefficient (*B*)	95% CI lower limit–upper limit	Standard partial regression coefficient (*β*)	*p*
**0.19**	Male: 0/female: 1	−17.3	−48.4 to 13.7	−0.11	0.27
	Age	−0.06	−1.4 to 1.2	−0.01	0.92
	Disease status (CR/PR: 0, SD/PD: 1)	−4.6	−39.5 to 30.2	−0.03	0.79
	ΔHb	14.6	1.23 − 28.1	0.24	0.03
	m‐CRS grade (mild: 0, moderate/severe: 1)	−37.3	−71.0 to −3.7	−0.25	0.03
	ICANS (−: 0, +: 1)	−56.0	−111.0 to −0.92	−0.22	0.04

Abbreviations: CI, confidence interval; CR, complete response; CRS, cytokine release syndrome; Hb, hemoglobin; ICANS, immune effector cell‐associated neurotoxicity syndrome; PD, progressive disease; PR, partial response; SD, stable disease.

Bold value statistically significant *p* < 0.05.

## 4 DISCUSSION

In this study, we implemented rehabilitation interventions for patients undergoing CAR‐T‐cell therapy and assessed changes in exercise tolerance before and after treatment. Our findings revealed that exercise tolerance was less likely to become impaired in CAR‐T‐cell therapy patients undergoing appropriate rehabilitation intervention. Moreover, a significant outcome of our study was its recognition of post‐CAR‐T‐specific complications, moderate/severe CRS, and ICANS, as key factors influencing the decline (or delayed recovery) in exercise tolerance after treatment, while other factors including sex, age, disease status were not significant risk factors for reduced exercise tolerance.

First, we checked pre‐CAR‐T treatment 6MWD and found that the median was as low as 450 m, which was lower than that observed in studies involving allogeneic hematopoietic stem cell transplant patients [12, 13]. Correlation analyses revealed that disease status (as indicated by disease status or LDH) and HCT‐CI was significantly associated with lower 6MWD. These results are supported by various findings or patient characteristics of our cohort. Indications for CAR‐T‐cell therapy primarily include refractory and relapsed patients [14, 15], and actually, most patients in this study had undergone multiple rounds of chemotherapy, including transplantation. Previous investigations comparing physical function before and after chemotherapy have consistently reported a gradual decline in physical capacity during treatment [16]. Moreover, poorly controlled disease status may induce chronic systemic inflammation leading to a state of cancer cachexia [17]. Our results of low 6MWD and associated factors are consistent with these studies, although the number of prior lines or courses of chemotherapy was not significant in this study, probably due to the small number of patients. Assessing physical function in patients before CAR‐T‐cell therapy and considering interventions to enhance exercise tolerance during the pretreatment phase may significantly optimize therapeutic outcomes.

Next, we analyzed the change of exercise tolerance before and after CAR‐T treatment. We confirmed that the majority of CAR‐T‐cell therapy patients under rehabilitation intervention exhibited only a minor decrease (or rather recovery) in 6MWD throughout treatment. These data partially prove the general hypothesis that CAR‐T‐cell therapy, requiring less intense pretreatment and other procedures, is less invasive than conventional auto‐ or allogeneic transplantation, where 6MWD deteriorates significantly, even with appropriate rehabilitation intervention. Moreover, we found that this result was parallel in each subgroup of age (younger or older), suggesting that CAR‐T‐cell therapy may be applicable to older patients when examined from the perspective of physical function. In our cohort, the maximum age was 75 years at the time of infusion.

After reviewing the whole trend of 6MWD, focusing on pre‐CAR‐T treatment variables, we then focused on post‐treatment CAR‐T‐specific adverse events and found that CRS and ICANS can negatively impact Δ6MWD. With recent advances in clinical management, including prompt response to tocilizumab and corticosteroid administration, the incidence of grade 2 or higher CRS has declined, whereas milder cases (grade 1) have become more prevalent. “Severity” of ASTCT grade 1 CRS varies widely, so our institution subdivided CRS into grades 1a and 1b, based on duration of CRS‐related symptoms [8]. Using that modified score, this study revealed that severe CRS as well as “moderate” CRS (grade 1 but longer duration of fever), and a decrease in Hb after CRS are risk factor for decrease or delayed recovery in exercise tolerance. This novel finding shows that long‐lasting CRS is significantly deleterious for patient physical status. Biologically, fever is a key factor leading to reduced physical activity and a heightened sense of fatigue [18]. In addition, prolonged fever can cause transient yet systemic inflammation and decreased oral intake, resulting in a hypercatabolic state [19, 20]. Moreover, a negative correlation between Δ6MWD and fever duration, as a continuous variable, supports the rationale for subdividing CRS into grades 1a and 1b, as a more sensitive identification of reduced exercise tolerance in patients classified as mild by conventional ASTCT standards. It is likely that the impact on exercise tolerance was greater in patients with an illness severity greater than grade 2, as these patients are subject to more intensive therapeutic management, including oxygen administration and other supportive measures. Additionally, a decrease in Hb following CAR‐T therapy was identified as a factor influencing Δ6MWD. Typically, a reduction in Hb triggers an increase in heart rate to maintain adequate oxygen supply to tissues, which may heighten subjective feelings of breathlessness. These Hb fluctuations during treatment could have impacted Δ6MWD. The severity of CRS is associated with cytopenia [8], and both CRS and the associated cytopenia were found to be significant factors affecting Δ6MWD.

Correlation with ICANS and post‐CAR‐T exercise tolerance is also newly revealed in this study. Decreased physical activity during ICANS might have exerted a greater influence than treatment of ICANS (corticosteroid administration), because corticosteroid use influences exercise tolerance typically only after a month.

Disease status and LDH were associated with pretreatment 6MWD, but were not significantly associated with Δ6MWD in our analysis; however, these factors were risk factors of CRS [21, 22, 23, 24, 25], and it is imperative to develop tailored rehabilitation strategies according to CRS severity risk factors, as well as pre‐CAR‐T 6MWD values.

This study covers comprehensive analyses of exercise tolerance before and after CAR‐T treatment, but has several limitations. First, this is a single‐center study with a limited number of cases. Therefore, further investigation is necessary to validate our results. Second, our study did not precisely evaluate the impact of rehabilitation itself on CAR‐T‐cell therapy patients, because all patients received the same rehabilitation intervention (no cohort without rehabilitation). To ascertain the effect of rehabilitation, a study comparing patients with or without rehabilitation, or different types of rehabilitation is necessary.

In conclusion, this study demonstrates that CAR‐T‐cell therapy patients undergoing rehabilitation exhibit a lower likelihood of developing exercise intolerance throughout the treatment phase. This potentially broadens the age criterion for CAR‐T‐cell therapy eligibility, based on exercise tolerance. Nevertheless, the severity of CRS and the incidence of ICANS could diminish exercise tolerance during treatment. Therefore, tailored intervention strategies should be developed for these patients in clinical practice.

## AUTHOR CONTRIBUTIONS

Ryota Hamada and Yasuyuki Arai designed the study, reviewed and analyzed data, and wrote the paper; Toshio Kitawaki, Naokazu Nakamura, Momoko Nishikori, Junya Kanda, Chisaki Mizumoto, Kouhei Yamashita, and Akifumi Takaori‐Kondo interpreted data and revised the manuscript; Masanobu Murao, Michiko Matsushita, Junsuke Miyasaka, Tsugumi Asano, and Ryosuke Ikeguchi contributed to the data collection and provided critiques on the manuscript.

## CONFLICT OF INTEREST STATEMENT

Momoko Nishikori received research grant from SymBio Pharmaceuticals Limited. The other authors declare no conflicts of interest.

## ETHICS STATEMENT

The study was approved by the pertinent ethics committee in Kyoto University (approval number: R‐0715).

## PATIENT CONSENT STATEMENT

All patients consented to participate in this study.

## CLINICAL TRIAL REGISTRATION

The authors have confirmed clinical trial registration is not needed for this submission.

## Data Availability

Data that support findings of this study are available from the corresponding author upon request.
